# Health behaviors influencing depressive symptoms in older Koreans living alone: secondary data analysis of the 2014 Korean longitudinal study of aging

**DOI:** 10.1186/s12877-018-0882-4

**Published:** 2018-08-20

**Authors:** Heejung Kim, Sooyoung Kwon, Soyun Hong, Sangeun Lee

**Affiliations:** 1 College of Nursing, Yonsei University, Seoul, South Korea; 2 Mo-Im Kim Nursing Research Institute, Yonsei University, Seoul, South Korea

**Keywords:** Older adult, Health behavior, Depressive symptoms, Living arrangement, Moderation, Korean longitudinal study of aging, Secondary data analysis

## Abstract

**Background:**

Geriatric depression is a societal problem, specifically in those living alone in Korea. This study aims are to investigate (1) how sociodemographic factors, health status, and health behaviors are differently associated with depressive symptoms in older Koreans living alone compared to those living with others and (2) how living arrangements attenuated or strengthened the associations between four types of health behaviors and depressive symptoms.

**Methods:**

This secondary data analysis was conducted using data from the 2014 Korean Longitudinal Study of Aging. A structured survey assessing sociodemographic factors, health status, and health behaviors was conducted with people aged 65 or older who lived alone (*n* = 1359) and living with others (*n* = 2864). A multiple linear regression with interaction terms was conducted between mean-centered health behaviors and the status of living alone. All statistical analyses were performed using SPSS Statistics 23.0, and the two-tailed level of significance was set at 0.05.

**Results:**

Those living alone reported higher levels of depressive symptoms than those living with others (*M*_*diff*_ = 2.129, *SE* = 0.005, *p* <  0.001). The variance of depressive symptoms explained by 13 variables was 18.1% for those living alone compared to 23.7% for those living with others. Compared to health behaviors, sociodemographic factors and health status more explained depressive symptoms, specifically with psychiatric disorders, pain, and impaired functionality as risk factors. Smoking, alcohol abstinence, physical inactivity, and social inactivity were associated with more depressive symptoms. Living arrangements moderated the association between depressive symptoms and each health behavior, except for physical inactivity (all *p* values < 0.001).

**Conclusions:**

Older Koreans living alone were exposed to different risk factors for depressive symptoms compared to those living with others. Non-modifiable sociodemographic and health status factors were highly associated with depressive symptoms relative to health behaviors; thus, it is important to conduct early assessment and classification of vulnerable subgroups regarding geriatric depression. Specific assessment instruments should be prepared in practice according to living arrangements among older Koreans. Targeted interventions are essential to addressing living arrangements and modifying health behaviors to reduce smoking, alcohol consumption, and social inactivity, specifically in those living alone.

## Background

Depression is one of the most prevalent mental health problems and is associated with general health and quality of life, specifically in late adulthood [1–3]. Most older adults seem to be exposed to a high risk of depression because they experience a series of losses, such as the death of significant others, retirement, or health problems [4–6]. Bereavement of a loved one results in social isolation and loneliness, which are significant risk factors for poor mental health and low quality of life [7, 8]. Some studies have reported that older adults experience a relatively lower socioeconomic status (SES) than that prior to retirement, which negatively affects mental health [3, 6]. Both social support and social strain differently mediate the relationship between increasing loneliness and decreasing well-being in retired older adults [6, 7]. Previous studies have investigated the diverse types of risk factors related to mental health, and living arrangement is one of the most important factors related to the complex nature of multidimensional vulnerability to depression in late adulthood [9–11].

Living alone influences mental health in the older adult population. Compared to those living with others, older adults living alone reported higher levels of depressive symptoms in several countries, such as Japan, Singapore, Taiwan, and the United States [8–10, 12]. In general, 20–30% of older Asians living alone reported significant depressive symptoms, a ratio that is significantly higher than the 12–18% reported among those living with others [8, 9]. In addition, older Americans living alone reported significantly more depressive symptoms (mean = 4.22, standard deviation [*SD*] *=* 4.56) than those living with a family member (mean = 3.36, *SD* = 3.71, *p* <  0.05) [10]. Similarly, up to 41% of older Koreans living alone reported depressive symptoms, which was significantly higher than up to 30% of those living with others [13, 14]. Oh and colleagues also reported that older Koreans living alone had a higher prevalence of depressive symptoms than those with any other type of living arrangement, such as living with or without a spouse in either an extended or a nuclear family [15].

Korean researchers, clinicians, and policy makers have paid attention to the vulnerability to depression in older adults living alone for several reasons. South Korea is the fastest-aging country among the Organization for Economic Cooperation and Development (OECD) countries, and Korea is also experiencing a rapid increase in one-person households [16, 17]. Older adults living alone comprised 23.0% of the aging population in 2014 [18], a dramatic increase from 13.6% in 1994 [18]. The number of people aged at least 65 years old or older living alone was estimated to be approximately 1.38 million in 2015; the number is estimated to increase to 3.43 million over the next 20 years [19]. The absolute and relative proportions of those living alone in the population mean that a new health care system must respond to the aging one-person household, as the family caregiving system has weakened in Korea due to changes of traditional and cultural perspectives on senior care [13].

In addition, older Koreans are less likely to receive appropriate treatment or perform self-management, despite high levels of depressive symptoms and related mental health problems [13]. In general, depression in older adults is under-reported and under-treated because older adults think that depression is part of the aging process [3, 20]. Specifically, older Korean adults have negative and passive views regarding mental health treatment. One study showed that only 20.9% of participants intend to use mental health services, although almost half of the subjects (49.3%) had issues with depression [21]. Because depression is more costly than other chronic physical diseases [3], untreated depression poses an increased individual and societal burden, influencing morbidity in conjunction with other chronic diseases and influencing mortality secondary to suicide [3, 22, 23]. The Global Burden of Disease Study 2010 identified depressive disorders as the second leading cause of long-term disability, designating it a major public health priority [22]. Older Korean adults are one of the most vulnerable populations to suicide; the suicide rate of older adults in South Korea has increased five-fold during the past two decades (approximately 70 people per every 100,000 in 2014), corresponding to the highest rate among the OECD countries [24].

However, there is limited information for understanding geriatric depression in older adults living alone and for developing specific prevention, detection, and treatment plans. Previous studies have shown that individual characteristics, such as being female, having poor self-rated health, and having impairments in both activities of daily living (ADLs) and instrumental activities of daily living (IADLs), are associated with depression in those living alone [10, 12, 14]. Although geriatric depression has great heterogeneity across people’s life spans and individual characteristics [3], few studies have conducted subgroup analyses to examine specific risk factors, particularly regarding different living arrangements [9, 12]. Moreover, these identified factors are more likely to be non-modifiable; therefore, it is difficult to improve the situation using individual efforts for the purposes of health promotion.

In this study, we focus on daily lifestyle health behaviors, which are considered the key components of self-management in disease prevention, treatment options, and physical and mental health [25, 26]. Similar to physical health, mental health status is considered to be influenced by health behaviors, such as alcohol consumption, smoking, and physical and social inactivity. It is well established in the literature that exercise is an effective intervention for improving mental health and well-being in later life [27–29]. Social isolation due to living alone and having less frequent contact with significant others is associated with a higher incidence of depression among older adults [9, 12, 14]. However, it is difficult to draw a concrete conclusion about how smoking and alcohol consumption affect depression in older adults. For example, a multisite cohort study showed that older adults with depression were likely to be alcohol abstinent or less-than-moderate drinkers, regardless of smoking status. However, smokers at risk of high alcohol consumption reported a three-fold greater likelihood of being depressed than those who were alcohol abstinent [30]. In contrast, other studies reported non-significant relationships among alcohol consumption, smoking, and depression in older adults [9, 14]. Thus, more research is needed to understand the consistent patterns of multiple health behaviors among depressed older adults in diverse populations, specifically when considering living arrangements [3].

The aim of this study was to examine the differences in the factors associated with depressive symptoms in older Koreans, specifically focusing on health behaviors moderated by living alone. The following research questions were proposed:

1. What is the difference in the sociodemographic factors, health status, and health behaviors of those living alone compared with those living with others?
2. How are sociodemographic factors, health status, and health behaviors associated with depressive symptoms in older adults with different living arrangements?
3. How does living arrangements differently moderate the relationships between depressive symptoms and four specific health behaviors after controlling for sociodemographic factors and health status?

## Methods

### Design

This was a cross-sectional correlation study with a secondary data analysis.

### Description of the primary data source and procedure for data collection

The primary data for this secondary data analysis were the Korean Longitudinal Study of Aging (KLoSA) collected in 2014 and released in 2015 by the Korea Employment Information Service [31]. The KLoSA is a nationally representative longitudinal study aimed at investigating the health and social welfare information of people aged 45 years or older in South Korea. To understand the selected topic with the most current trend, we chose the most recent set of KLoSA data available for public use.

Since 2006, the KLoSA panel survey has been conducted biennially by trained interviewers using a computer-assisted personal interviewing method in which interviewers read questions to respondents from screens and immediately enter their responses. The survey is conducted with identical content for the same respondents from the first to fifth waves to collect observations at multiple times [32]. The survey targeted the middle-aged and older population nationwide, except in island areas. The sample was randomly selected using a multistage, stratified probability sampling design based on geographical areas and housing types across the nation. At the first data collection, 10,254 individuals in 6171 households participated in the interview, and 7029 subjects remained in 2014, which represented 72.8% of the original respondents [31].

### Samples of secondary data analysis

Among the 7029 participants in the 2014 KLoSA dataset, 4223 eligible subjects were included in this study after excluding those aged younger than 65 years old (*n* = 2803) and those with incomplete reports (*n* = 3) for the Center for Epidemiological Studies-Depression Scale short-form 10 item (CES-D10). Older adults living alone were defined as individuals aged 65 years old or older in 2014 who reported only one member in a single generation household (*n* = 1359). The other comparison group included older adults who were 65 years old or older in 2014 and who lived with any members in the same house (*n* = 2864).

### Measures

Depressive symptoms were measured using the Boston version of the CES-D10 in the 2014 KLoSA survey. The CES-D, which was developed by Radloff (1977), is a screening tool used to assess the depressive symptoms experienced during the most recent week. Each item was measured on a 4-point Likert scale (0 = *very rarely* or *less than once a day*; 3 = *almost always* or *5–7 days during the past week*). After two items were reversely recoded to calculate a total CES-D10 score, a composite score was generated by summing ten items. Higher scores indicated more depressive symptoms (range: 0–30). In this study, the Cronbach’s alpha coefficient for the CES-D10 was 0.848 in total, 0.841 in those living alone, and 0.853 in those living with others. We used CES-D scores as a continuous variable in line with the methodology employed in previous studies [12, 14, 33] because (1) we aimed to compare our findings to the previous studies’ findings, (2) CES-D was developed to offer screening rather than as a comprehensive diagnostic test, and (3) inconsistency arises when using a specific cutoff to differentiate depressed and non-depressed groups in different populations [28].

Sociodemographic factors were included as categorical variables: age, gender, education level, and SES. Considering normative information about older Koreans, the three age groups were categorized as young–old (65–74; coded as 0), old–old (75–84), and oldest–old (85 and over). Gender was dichotomized as female and male (coded as 0). Education level was classified into no school education (coded as 0), elementary school, middle school, high school, and college or above. Self-perceived SES was classified into 3 groups: low, middle (coded as 0), and high.

Self-rated pain was dichotomized as 0 = *none* and 1 = *any pain experienced in one or more body parts*. Psychiatric illness was either diagnosed by a physician or self-reported as having any psychiatric symptoms (coded as 1) and was otherwise coded as 0. A self-rated health status was assessed using the following question: “In general, would you say your health is excellent, very good, good, fair, or poor?” Higher scores indicated perceptions of poorer health status, while the good group was coded as 0. Chronic medical conditions were accounted for in the number of self-reported diagnoses, such as hypertension, diabetes mellitus, any type of cancer, chronic lung disease, liver disease, heart disease, cerebral vascular disease, or rheumatoid arthritis, which are the most frequent conditions reported by older Koreans [34]. Each condition was reported based on 0 = *absent* and 1 = *present*, based on symptoms experienced and medical diagnoses. The impaired functionality of older adults was measured based on LaPlante’s expanded ADLs and IADLs scales, including grocery shopping, getting to places, performing light housework, preparing meals, bathing, getting outside, walking, dressing, managing money, transferring, managing medications, using the restroom, using the telephone, and eating [35]. Each item was recoded as 0 = *completely independent* or 1 = *partial/completely dependent*, and the sum of the scores for the 14 items was used in the data analysis. Higher scores indicated that older adults were more dependent on others when performing ADLs and IADLs in daily life. In this study, the Cronbach’s alpha coefficient for the LaPlante’s ADLs and IADLs was 0.963 in total, 0.958 in those living alone, and 0.967 in those living with others.

Smoking and alcohol use were dichotomized as 0 = *non-smoker* or *non-drinker* and 1 = *current smoker* or *active alcohol user*. The physically active group was defined as those who exercised more than once a week (coded as 0); the others were coded as 1. A lack of social participation was coded as 1, while participation in any social activities, such as attending religious gatherings, meeting friends, volunteering, or joining hobby clubs, was coded as 0.

### Analysis

We calculated the weighted proportions, means, and SDs of sociodemographic characteristics, health status, and health behaviors between older Koreans living alone and those living with others. The missing data for all the variables tested were 0.002%. We did not complete missing data imputation because the total amount of missing data was less than 5.0%, and a missing data analysis found that such missing data occurred at random [36]. All analyses were conducted by applying population weights, which were calculated based on the following: (a) a two-staged stratified sampling probability due to the design effect; (b) the non-response-adjusted weight to reduce the non-response bias; and (c) the benchmark weight, reflecting changes in the general distribution of the total population of the Republic of Korea in 2014 [31].

For preliminary comparison, multivariate linear regression models were used to evaluate the relationship of depressive symptoms with sociodemographic characteristics (Block 1), health statuses (Block 2), and health behaviors (Block 3) in older adults living alone and those living with others. Assumptions of the multivariate linear regression analyses (univariate and multivariate normality, linearity, homoscedasticity, and diagnostic testing for multicollinearity and independence of errors) were met [36].

A multiple linear regression with an interaction term was conducted to assess the associations between health behaviors and depressive symptoms moderated by living arrangement. Age, gender, and low SES were included as covariates in Step 1. The four health behaviors were entered in Step 2 to test each main effect on depressive symptoms over and beyond three covariates, while living arrangement was entered in Step 3. Finally, all the interaction terms between each health behavior and living arrangement were entered in Step 4 to examine the moderating effects. We decided not to include health status variables, which have spurious associations simultaneously with both health behaviors and depressive symptoms. Four health behaviors and living arrangements were mean-centered. A significant standardized regression coefficient and change in *R*^2^ for the interaction term indicated a significant moderation effect [37]. All statistical analyses were performed using IBM SPSS Version 23.0, and the two-tailed level of significance was set at 0.05.

## Results

### Sociodemographic and health-related characteristics of the sample

Table 1 presents the sociodemographic and health-related characteristics (health status and behaviors) of the weighted sample. The mean age of older Koreans in this study was 73.81 (*SD* = 6.68) years, and 58.1% were females. Half of the respondents had either no education (22.1%) or had only attended elementary school (34.8%). Most of them identified their SES as middle (45.3%) or low (52.4%). Almost half of the respondents rated their health as good (44.2%), followed by fair (30.5%), very good (15.8%), poor (8.9%), and excellent (0.6%). On average, they experienced 0.98 functional impairments based on LaPlante’s ADL/IADL (*SD* = 2.92), 0.14 chronic diseases (*SD* = 0.41), and a score of 7.62 on the CES-D10 (*SD* = 5.60). Approximately 5.6% of them were diagnosed with or self-recognized a psychiatric illness. The percentage of those experiencing any pain was 71.3%. The percentage of people who smoked or drank alcohol was 10.2% and 27.2%, respectively, while 32.2% of the participants regularly exercised more than once a week. More than 70% of them participated in some type of social activities.

**Table 1:** Group differences in sociodemographic and health-related characteristics

Variables	Overall	Those living alone mean (SD) or weighted %	Those living with others mean (SD) or weighted %	*p* value
Age	73.81 (6.68)	75.12 (6.68)	72.99 (6.54)	< 0.001
LaPlante ADL/IADL impairment	0.98 (2.92)	1.01 (2.87)	0.97 (2.95)	< 0.001
Number of chronic diseases	0.14 (0.41)	0.15 (0.43)	0.14 (0.39)	< 0.001
CES-D10 scores	7.62 (5.60)	8.52 (5.52)	7.05 (5.59)	< 0.001
Gender^a^				
Female	58.1	76.6	46.5	< 0.001
Education				
No school attended	22.1	30.0	17.1	< 0.001
Elementary school	34.8	37.2	33.4	
Middle school	16.7	14.3	18.2	
High school	17.9	12.8	21.1	
College or above	8.5	5.7	10.2	
Socioeconomic status				
High	2.3	1.7	2.7	< 0.001
Middle	45.3	37.7	50.0	
Low	52.4	60.6	47.3	
Self-rated health				
Excellent	0.6	0.4	0.8	< 0.001
Very good	15.8	14.0	16.9	
Good	44.2	39.5	47.2	
Fair	30.5	36.6	26.7	
Poor	8.9	9.4	8.5	
Pain^a^				
Present	71.3	74.9	69.0	< 0.001
Psychiatric illness^a^				
Diagnosed	5.6	6.4	5.1	< 0.001
Smoking status^a^				
Current smoker	10.2	7.8	11.7	< 0.001
Alcohol use^a^				
Active drinker	27.2	21.3	30.9	< 0.001
Regular exercise^a^				
More than once a week	32.2	31.4	32.7	< 0.001
Social activity ^a^				
Participating	71.1	73.0	68.1	< 0.001

*SD* Standard deviation; *CES-D10* the Center for Epidemiologic Studies Depression Scale-10 items, *ADL/IADL* Activities of daily living/Instrumental activities of daily living

^a^, the variable was dichotomized

### Group comparison of characteristics by living arrangements

Most of the characteristics of participants living alone were significantly different from the characteristics of those living with others. Those who lived alone were slightly older (mean difference [*M*_*diff*_] = 2.129, standard error [*SE*] = 0.005), had more functional impairments (*M*_*diff*_ = 0.036, *SE* = 0.002), had higher CES-D10 scores (*M*_*diff*_ = 1.473, *SE* = 0.005), and lived with more diagnosed diseases than those living with others (all *p* values < 0.001). Compared to those living with others, the majority of those living alone were female, educated at a less than middle school level, had a lower SES or poorer health status, and experienced more pain (all *p* values < 0.001). People who lived alone were less likely to smoke, drink alcohol, and exercise regularly than those living with others (all *p* values < 0.001). However, those living alone participated in more social activities (73.0%) than those living with others (68.1% and 43.4%, respectively; all *p* values < 0.001).

### Multivariate linear regression analyses in two groups

The results of the multivariate linear regression using the CES-D10 score as the dependent variable and the 13 factors in three blocks are shown in Table 2. The separate models between older adults living alone and those living with others were statistically significant and explained 18.1% and 23.7% of the variance in the depressive symptoms, respectively. In the model of those living alone, the block of health status explained 10.12% of the variance, followed by the sociodemographic block (7.12%) and the block of health behaviors (0.82%; *F* = 28,354.14, *p* <  0.001). In the model of those living with others, the block of health status explained 16.91%, followed by 5.69% for the sociodemographic block and 1.06% for the block of health behaviors (*F* = 64,147.47, *p* <  0.001).

**Table 2:** Comparison of multivariate linear regression models between two groups

Variables	Those who live alone				Those who live with others			
	B	SE	β	Δ R^2^ change	B	SE	β	Δ R^2^ change
Constant	5.454	0.015			4.204	0.012		
Block 1: Sociodemographic factors				0.071^***^				0.057^***^
Age (ref., 65–74)								
75–84	0.496^***^	0.007	0.044		0.144^***^	0.006	0.012	
Over 85	0.741^***^	0.013	0.039		−0.195^***^	0.011	− 0.009	
Gender (ref., male)								
Female	0.073^***^	0.009	0.006		−0.489^***^	0.006	−0.044	
Education level (ref., no school)								
Elementary school	−0.008	0.008	−0.001		−0.015^*^	0.008	−0.001	
Middle school	−0.430^***^	0.011	−0.027		0.066^***^	0.009	0.005	
High school	0.904^***^	0.012	0.055		−0.110^***^	0.009	−0.008	
College and above	−0.465^***^	0.017	−0.020		−0.066^***^	0.011	−0.004	
Socioeconomic status (ref., middle)								
High	0.822^***^	0.025	0.019		0.990^***^	0.016	0.029	
Low	1.139^***^	0.007	0.101		0.808^***^	0.005	0.072	
Block 2: Health status				0.101^***^				0.169^***^
Self-rated health status (ref. good)								
Excellent/Very good	−0.762^***^	0.010	−0.049		1.037^***^	0.007	0.071	
Fair/Poor	2.214^***^	0.008	0.201		2.395^***^	0.006	0.205	
Numbers of current chronic diseases	−0.234^***^	0.008	−0.018		−0.173^***^	0.006	−0.012	
Psychiatric illness	2.463^***^	0.013	0.109		3.431^***^	0.012	0.136	
Active pain	0.787^***^	0.008	0.062		1.545^***^	0.006	0.128	
LaPlante ADL/IADL impairment	0.221^***^	0.001	0.114		0.383^***^	0.001	0.202	
Block 3: Health behaviors				0.008^***^				0.011^***^
Active smoking	1.337^***^	0.013	0.065		−0.070^***^	0.008	−0.004	
Alcohol drinking	−0.672^***^	0.009	−0.050		−0.958^***^	0.006	−0.079	
Physical inactivity	−0.023^**^	0.007	−0.002		0.103^***^	0.006	0.009	
Social inactivity	0.678^***^	0.007	0.057		1.015^***^	0.006	0.081	
Total adjusted R^2^				0.181^***^				0.237^***^

Dependent variable = Total score of the Center for Epidemiologic Studies Depression Scale-10

*ADL/IADL* Activities of daily living/Instrumental activities of daily living

^*^ *p* <  0.05, ^**^ *p* <  0.01, ^***^ *p* <  0.001

Table 2 shows significant factors associated with depressive symptoms in both models. Specifically, psychiatric illness, active pain, and impaired functionality of ADL/IADL consistently increased depressive symptoms in both groups; however, a stronger impact was observed in those living with others than in those living alone. The levels of education showed a U-shaped relationship with depressive symptoms in both groups; however, greater variability among educational subgroups was observed in those living alone than in those living with others. In the group living alone, those older than 85, women, and those currently smoking reported increased CES-D10 scores compared to the opposite result being found in those younger than 85, males, and non-smokers. In addition, the excellent health status of those living with others was associated with higher CES-D10 scores, while the reverse relationship was found in those living alone.

### Moderation effects of living arrangement on the relationship between depressive symptoms and health behaviors

The main and interaction effects of living arrangement on the relationship between depressive symptoms and four health behaviors are presented in Table 3. Smoking, alcohol abstinence, physical inactivity, and social inactivity were associated with higher levels of depressive symptoms, controlling for age, gender, and low SES. Living arrangement was positively associated with depressive symptoms, indicating a main effect on depressive symptoms. Adding interaction variables during the final stage of the analysis revealed different patterns for each health behavior.

**Table 3:** Result of multiple linear regression with interaction term

	Variables	Adj. R^2^	Δ R^2^	B	SE	β	*p* value
Step 1	Sociodemographic factors	0.068^***^	0.068^***^				
	Aged at 75-84^a^			0.807	0.005	0.068	< 0.001
	Aged over 85^a^			1.629	0.008	0.076	< 0.001
	Gender^b^			0.053	0.005	0.005	< 0.001
	Low socioeconomic status^c^			1.427	0.004	0.127	< 0.001
Step 2	Health behaviors	0.104^***^	0.035^***^				
	Active smoking			0.371	0.007	0.020	< 0.001
	Alcohol drinking			−1.307	0.005	−0.104	< 0.001
	Physical inactivity			0.614	0.005	0.051	< 0.001
	Social inactivity			1.957	0.005	0.158	< 0.001
Step 3	Living arrangement	0.109^***^	0.005^***^	0.916	0.005	0.080	< 0.001
Step 4	Active smoking × Living arrangement	0.112^***^	0.003^***^	0.199	0.002	0.035	< 0.001
	Alcohol drinking × Living arrangement			0.081	0.002	0.015	< 0.001
	Physical inactivity × Living arrangement			−0.001	0.002	0.000	0.678
	Social inactivity × Living arrangement			−0.180	0.002	−0.034	< 0.001

^a^Referent group = aged at 65–74; ^b^Referent group = male; ^c^Referent group = middle level of socioeconomic status; ^***^ *p* < 0.001

**Fig. 1:**
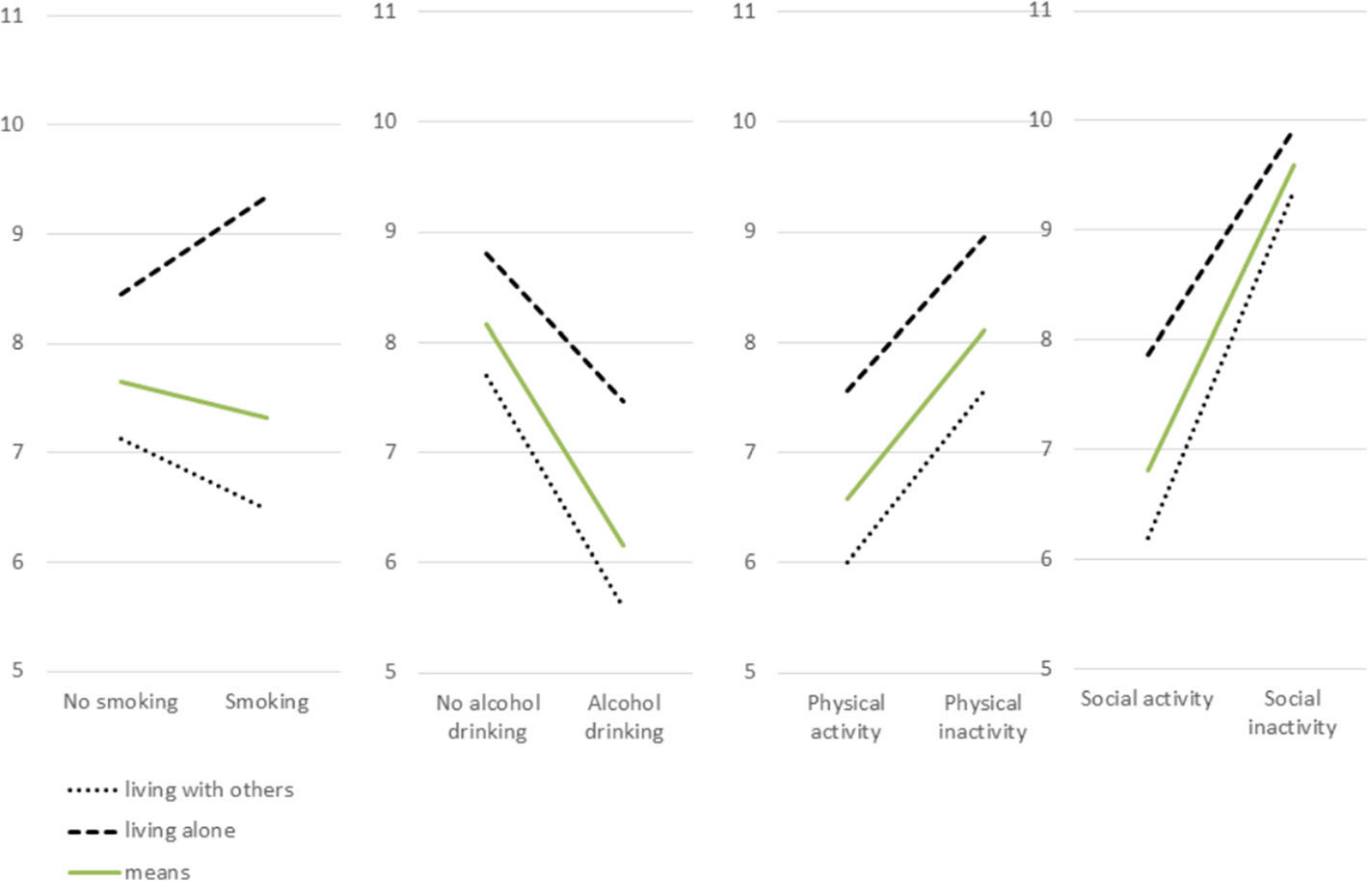
Mean difference of depressive symptoms in each health behavior group moderated by living arrangement

Living arrangement showed significant interaction with smoking, alcohol consumption, and social activity, while there was non-significant interaction with physical activity. The association between active smoking and depressive symptoms was attenuated by taking living arrangement into consideration. However, the association of depressive symptoms with alcohol drinking and social isolation became stronger when considering living arrangement, specifically regarding living alone. The mean level of depressive symptoms for each significant moderator stratified by the living arrangement is illustrated in Fig. 1.

## Discussion

This study provides important information about the unique factors of geriatric depression identified among older adults living alone and those living with others, specifically focusing on the moderating effects of health behaviors. In general, older Koreans living alone reported higher levels of depressive symptoms than those living with others, which were similar to the findings of previous reports [12, 15, 38]. Our findings confirmed significant similarities and differences among factors influencing depressive symptoms between the two groups.

Our study findings showed inconsistent directions between smoking and depressive symptoms, depending on living arrangements. Older smokers living alone reported more depressive symptoms; however, older smokers living with others had fewer depressive symptoms. In contrast, alcohol abstinence was consistently associated with more depressive symptoms, similar to previous reports [14, 30]. Smoking and drinking alcohol are considered stress-reducing behaviors in adults [26]. Some studies in Western countries reported smoking and heavy drinking as being positively associated with depression [30, 39, 40]. However, some studies have reported a non-significant relationship between geriatric depression and drinking alcohol and smoking in different populations [9, 38, 41]. Interestingly, smoking and alcohol consumption show an inverse association with depressive symptoms in some Asian countries [14, 41, 42]. In Asian culture, smoking and alcohol consumption play a role in media when participating in social interaction. For example, 27.17% of older men and 4.63% of older women reported social drinking, which was associated with a decreased risk for depression [43]. Thus, this finding implies that health-promoting behavior should be understood in the context of culture to understand different patterns of risk factors reported by diverse types of cultural populations [44].

Physical inactivity was associated with more depressive symptoms, regardless of living arrangements. Several studies reported engaging in physical activity as being effective in reducing depressive symptoms among diverse types of older adults [28, 29, 45]. Exercise may provide opportunities to (1) alter or reduce negative thoughts in daily life, (2) learn a new skill dealing with stress responses, and (3) increase social contact outside of family systems [46]. Some previous studies have shown that older adults reported increased levels of cognition, quality of life, and well-being after receiving physically active interventions [27, 29]. In addition, older adults believe that physical activity could be a non-pharmacological treatment that is alternative to antidepressants for reducing depressive symptoms [26, 47]. A Cochrane Database of Systematic Review [48] reported that exercise moderately reduced depressive symptoms compared to no treatment, antidepressants, and psychological therapies. Thus, increasing physical activity for older adults is recommended due to high levels of acceptability, the prevention of polypharmacy, and the reduction of medication-adherence concerns, specifically for mild to moderate depression [49].

Social inactivity was associated with more depressive symptoms in general, similar to previous reports [14, 38]. In our study, socially inactive individuals do not participate in any activities during social gatherings, supportive groups, or organizational meetings. Social support is considered to be a protective factor for dealing with stressful life events experienced by older adults [9, 11]. Inadequate levels of perceived social interaction and support result in loneliness and in a lower quality of life, as well as poor mental health [11]. However, our study added more findings, indicating that the difference within groups was more notable in those living with others than those living alone. Providing adequate levels of social support may critically decrease depressive symptoms in older adults living with others [9, 11], although grown children are expected to live with their aging parents or to provide support through frequent visits in Asian countries [12]. When older adults living alone have less frequent contact with their family, they seek alternative sources of support from their neighborhood, religious group, or community [11, 12]. Thus, the Korean government operates senior centers that are specialized in caring for those living alone in the community. Community nurses working with those living alone provide a wide range of interventions or programs to enhance these clients’ social connectedness.

The group of older adults living alone showed some expected factors that were associated with more depressive symptoms, such as gender (i.e., women) [3, 14, 15] and advanced age [9, 15, 38], in this study. However, unexpected findings were observed in the group of older adults living with others; namely, men and those aged 75–84 years reported higher scores of depressive symptoms than those younger than 85 years, men, and non-smokers. It is difficult to make a directional conclusion because few studies have reported gender differences in factors associated with depressive symptoms among older Koreans [14, 50]. In general, older men showed lower levels of help-seeking behaviors for informal support than older women when dealing with depressive symptoms [47]. However, both perceived that depression and help-seeking behaviors are greatly influenced by culture; therefore, caution is required when interpreting this finding, and more evidence is necessary to understand older men’s depression in Korea.

Similar to previous studies, our findings confirmed that more depressive symptoms were reported in older adults with low SES [1, 38, 51]. In addition, a significant association between education and depressive symptoms was observed in educated individuals in both groups [1, 15]. Because of the high correlation between education levels and income levels, those factors simultaneously affect the levels of SES among Koreans. Those with low SES have a higher chance of depression due to repeated stress from negative life experiences, such as helplessness, poor living conditions, and lack of coping resources [52]. In our study, when we compared subgroups based on education level, those with more depressive symptoms were most likely to have a mid-level education, followed by the lowest and then the highest education. Although educational attainment is associated with stress appraisal when dealing with psychological distress and physical illness [52], this U-shaped relationship between education level and depressive symptoms was not fully explained in previous reports about Korean populations [13–15]. Further investigation is required to develop tailored interventions based on different levels of education.

Unexpectedly, the number of chronic diseases was weakly associated with depressive symptoms, dissimilar to previous studies [1, 3, 15, 38, 51, 53]. Previous studies showed that a combination of multiple chronic disease diagnoses has a limited ability to predict depressive symptoms, although each diagnosis, such as diabetes, stroke, heart disease, and head trauma, uniquely increases depression scores [14, 38]. Instead, psychiatric illness is a consistent factor that increases depressive symptoms, regardless of living arrangements, similar to the previous report [54]. The status of psychiatric illness may affect the age of onset, the number of lifetime episodes, somatic symptoms, and comorbidities contributing to or resulting from the psychopathology of geriatric depression [3]. Thus, future studies should focus on a specific disease group to differentiate the impacts of various diseases on depressive symptoms among older adults, given that each medical condition has a unique disease trajectory and socio-demographic characteristics [14, 38].

Poor health status, pain, and functional impairments were common factors that increased the depressive symptoms in both groups, similar to previous reports [3, 14, 53]. Those conditions are considered to be age-related factors necessary for maintaining independent living, which is highly associated with increased depressive symptoms [3, 55]. A nationwide survey of the Taiwan Longitudinal Study suggested that those living alone are sufficiently competent to handle their own daily living and manage their medical conditions [12]. However, as functionality decreases with age, an individual’s capacity of self-management and self-care mechanisms of health highly depends on impaired function that individual has suffered from chronic diseases [51]. In addition, those factors influence an individual’s presentation of atypical symptoms resulting from depression at different levels, which increase the likelihood of being under-detected and underestimated in depressed adults [38]. Thus, the early detection of those factors to differentiate risk factors and atypical symptoms is critically important to provide symptom-based interventions when co-residents are absent.

In clinical implication, depressive symptoms in older Koreans were mostly associated with sociodemographic factors and health status rather than health behaviors, regardless of living arrangement. It is important to assess both the depressive mood and relevant characteristics of older adults in general and to classify the vulnerable subgroups based on predisposing factors of depression. The screening instrument, such as the geriatric depression screening scale [56], is associated with symptoms rather than risk factors. Thus, identification of even non-modifiable factors is important to clearly define the most vulnerable group. In addition, targeted interventions should be designed to consider the unique characteristics and situations of older adults living alone when simultaneously modifying multiple health behaviors differently.

### Study limitations and future research

Most of the study’s limitations resulted from the study’s design, which consisted of a secondary data analysis with cross-sectional data. First, we were unable to include any variables that reflected the influence of the duration of living alone or any recent changes in living arrangements on depressive symptoms. Some research has shown that the impact of living alone is attenuated over time [14]; thus, further research should include variables differentiating between the short- and long-term effects of living alone to maximize the advantage of repeated measures in longitudinal surveys. Second, self-report instruments have limitations in terms of diagnosing major depressive disorder, requiring instant medical attention. Thus, further research must examine the proportion of those diagnosed with major depressive disorder medically to determine the appropriate clinical action for the most vulnerable subgroups of older adults. Third, we included limited numbers of variables associated with health behaviors, namely those available in the primary data. Previous research has shown significant relationships between geriatric depression and other variables, such as diet and sleep [9, 14]. Hence, we suggest that further studies include more extensive information on health behaviors associated with this topic. Health-lifestyle theories have emphasized integrated strategies to simultaneously modify multiple health behaviors for more effective and sustainable health promotion relative to single-behavior modification [26]. The distinct impact of each health behavior on depression is not identical to the cumulative effect of a combination of multiple activities [14]. Further research is required to confirm the best combination of health behaviors to reduce depression in older Koreans living alone, targeting both common and situation-specific depression components.

## Conclusion

Health behaviors were weakly associated with depressive symptoms relative to non-modifiable sociodemographic and health-status factors. However, different patterns between each health behavior and depressive symptoms were found in this study. Smoking, alcohol abstinence, and social inactivity were associated with more depressive symptoms moderated by living arrangements; however, physical inactivity was associated with depressive symptoms but was not moderated by living arrangements. Further research should focus on the different patterns of geriatric depression among diverse sociodemographic subgroups and specific types of health behaviors. Policy makers and clinicians should better prepare to understand sociodemographic and health-related characteristics to improve the mental health and quality of life of emerging older adults who live alone.

## Abbreviations

ADLs: Activities of daily living
CES-D10: the Center for Epidemiological Studies-Depression Scale short-form 10 items
IADLs: Instrumental activities of daily living
KLoSA: Korean Longitudinal Study of Aging
OECD: Organization for Economic Cooperation and Development
SES: Socioeconomic status
